# The Use of Chitin from the Molts of Mealworm (*Tenebrio molitor*) for the Removal of Anionic and Cationic Dyes from Aqueous Solutions

**DOI:** 10.3390/ma16020545

**Published:** 2023-01-05

**Authors:** Tomasz Jóźwiak, Urszula Filipkowska, Tadeusz Bakuła, Beata Bralewska-Piotrowicz, Konrad Karczmarczyk, Magdalena Gierszewska, Ewa Olewnik-Kruszkowska, Natalia Szyryńska, Bogdan Lewczuk

**Affiliations:** 1 Department of Environmental Engineering, University of Warmia and Mazury in Olsztyn, Warszawska St. 117a, 10-957 Olsztyn, Poland; 2 Department of Veterinary Prevention and Feed Hygiene, University of Warmia and Mazury in Olsztyn, Oczapowskiego 13 St., 10-718 Olsztyn, Poland; 3 Department of Physical Chemistry and Physicochemistry of Polymers, Nicolaus Copernicus University in Toruń, 7 Gagarina St., 87-100 Toruń, Poland; 4 Department of Histology and Embryology, University of Warmia and Mazury in Olsztyn, Oczapowskiego 13 St., 10-719 Olsztyn, Poland

**Keywords:** mealworm, chitin, sorption, dyes

## Abstract

The possibility of using chitin from the molts of an insect–ealworm (*Tenebrio molitor*) to remove anionic (RB5, RY84) and cationic dyes (BV10, BR46) from aqueous solutions was investigated. The scope of the research included, among others: Characteristics of chitin from mealworms (FTIR, SEM, pH_PZC_), the effect of pH on sorption efficiency, sorption kinetics (pseudo-first, pseudo-second order, intramolecular diffusion models) and the determination of the maximum sorption capacity (Langmuir and Freundlich models). The sorption efficiency of anionic dyes on chitin from mealworm was the highest at pH 2–3, and for cationic dyes at pH 6. The equilibrium time of sorption of anionic dyes was 240–300 min and for cationic dyes it was 180–240 min. The experimental data on dye sorption kinetics was best described by the pseudo-second order model. The maximum sorption capacity of chitin from the mealworm for the anionic dyes RB5 and RY84 was 121.15 mg/g and 138.55 mg/g, respectively, and was higher than with some carbon-based materials (literature data). In the case of cationic dyes, the sorption capacity of the tested chitin was lower and reached 3.22 mg/g and 59.56 mg/g for BV10 and BR46, respectively.

## 1. Introduction

Wastewater produced by the textile, tanning, and paper industries is particularly harmful to the environment. This is usually due to the high amounts of synthetic dyes used in the dyeing processes [1]. Color substances that have penetrated into the aquatic environment can significantly contribute to its degradation. Dyes dissolved in water restrict access to sunlight for aquatic plants and may inhibit the primary production process in water reservoirs. These substances also limit the diffusion of oxygen in water, which, combined with the loss of the autotrophs’ ability for photosynthesis, may lead to the risk of appearance of local anaerobic conditions [2]. Moreover, many dyes and their degradation products, such as aromatic amines, exert mutagenic and carcinogenic effects on aquatic organisms. In extreme cases, the presence of colored substances in natural waters may cause the collapse of the local water ecosystem [3,4]. The high risk of the aquatic environment degradation posed by the penetration of colored substances into it indicates the need for the implementation of effective and environmentally safe methods for color wastewater decolorization.

The currently used dyes are hardly biodegradable substances because of their complicated chemical structure. For this reason, the treatment of colored wastewater with traditional, biological methods, based on activated sludge or biological membrane technology, is usually ineffective [5]. Decolorization of wastewater is much more effective with the use of physicochemical methods, like precipitation methods, deep oxidation methods and membrane processes [6]. Precipitation methods, such as coagulation, ensure relatively quick removal of dye molecules from wastewater; however, these processes are also associated with wastewater salination and generation of large amounts of sludge, which is difficult to manage [7]. Decolorization of wastewater by deep oxidation (e.g., ozonation, reaction with NaOCl) effectively removes the color of most types of colored wastewater. However, the oxidation of dyes present in wastewater poses the risk of the accumulation of its toxic intermediates, such as aromatic amines, in the system [8]. In turn, the membrane methods for wastewater decolorization, such as reverse osmosis, are characterized by nearly 100% efficiency, regardless of the type of dyes present in colored wastewater [9]. However, reverse osmosis is extremely costly and involves a large loss of water during the process.

Many scientists have posited the sorption process to be both the most environmentally friendly and one of the best methods for wastewater decolorization [3]. During sorption, no sludge or any toxic compounds are formed in the wastewater. The cost of the process, as well as its efficiency, depends mainly on the type of sorbent used. The most widely used sorbents today are materials based on activated carbon. They show relatively high efficiency in binding dyes from wastewater [10], but are quite expensive. Therefore, cheaper alternatives are being sought.

Chitin is an interesting material showing very high sorption capacity towards dyes. It is a biopolymer that is the basic building component of fungal cell walls and arthropod exoskeletons. It may be obtained in industrial amounts from shells of sea crustaceans (shrimps and crabs), i.e., waste products of the seafood processing industry [11]. For this reason, this material is considered to be widely available and also relatively cheap. In recent years, however, the availability of raw materials for the production of chitin has decreased due to pharmaceutical companies, which buy them to produce dietary supplements (including chitin and glucosamine). These resources are scarce, especially in landlocked countries and in countries where shellfish consumption is low.

Insects may represent an alternative source of chitin [12]. Currently, there is an increasing interest in breeding insects for feed and food purposes. An example of such an insect is the mealworm (*Tenebrio molitor*). In 2021, the mealworm larvae were classified by the European Union as “novel food” [13]. New farms for breeding this insect are being built in many countries. The edible mealworm larvae shed about 15 times within 10 months. The molts left by the larvae contain up to several dozen percent of chitin in dry matter, which makes them a potentially good raw material for the production of purified chitin. The structure of the chitin sorbent obtained from the molts of mealworm may differ from the structure of the sorbent from shellfish shells, which results in differences in sorption properties.

This study’s goal was to investigate the sorption properties of chitin obtained from the molts of mealworms against the anionic dyes popular in the industry. Reactive Black 5 and Reactive Yellow 84, and cationic dyes: Basic Violet 10 and Basic Red 46.

## 2. Materials and Laboratory Equipment

### 2.1. Molts of Mealworm

The molts of mealworm came from the laboratory breeding of insects. The insects were fed with oatmeal, carrots, and apples. Cannibalism was not found in breeding animals.

### 2.2. Dyes

The study was performed with reactive anionic dyes popular in the textile industry (Reactive Black 5, Reactive Yellow 84) as well as cationic dyes (Basic Violet 10, Basic Red 46). The characteristics provided by the manufacturer are summarized in Table 1.

### 2.3. Chemical Reagents

- Hydrochloric acid (HCl)—37%—(demineralization of mealworm molts, pH correction of dye solutions);
- Sodium hydroxide (NaOH) > 99.9% (microgranules)—(deproteinization of mealworm molts, pH correction of dye solutions);
- Buffer solutions for calibrating the pH meter (pH 4 ± 0.05/pH 7 ± 0.05/ pH 10 ± 0.05).
- All chemical reagents used were purchased from POCH S.A., Gliwice, Poland, and were of p.a. (analytical) purity or higher.

### 2.4. Laboratory Eqiupment

- HI 110 pH meter (HANNA Instruments, Olsztyn, Poland)—for the measurement and correction of solutions pH;
- Laboratory shaker SK-71 (JEIO TECH, Daejeon, Korea)—for the process of sorption;
- Multi-Channel Stirrer MS-53M (JEIO TECH, Daejeon, Korea)—for dye sorption analyses;
- UV-3100 PC spectrophotometer (VWR spectrophotometers, VWR International LLC., Mississauga, ON, Canada)—for determination of the concentration of dye in solutions;
- FT/IR-4700LE FT-IR Spectrometer with single reflection ATR attachment (JASCO International, Tokyo, Japan)—for the preparation of sorbent’s FTIR spectra;
- Gemini VI (Micromeritics, Norcross, GA, USA)—for the measurements of porosity and surface area of the sorbent;
- Scanning Electron Microscope Gemini 450 (Carl Zeiss Microscopy, Jena, Germany)—for taking images of the sorbent’s surface morphology.

## 3. Methodology

### 3.1. Preparation of the Sorbent (CHM)

The molts of mealworm (30 g DM—dry mass) were introduced into a beaker (2000 mL capacity). Next, a 2 M HCl solution (1500 mL) was added to the beaker, and the content was mixed (until all molts were completely submerged). After 3 h of molts demineralization in hydrochloric acid, the obtained material was filtered and washed with deionized water on a laboratory sieve (with a mesh diameter of 0.5 mm). The demineralized molts were placed in a beaker (600 mL capacity) to which 500 mL of 2 M NaOH was then added. The beaker was placed on a magnetic stirrer with heating (150 r.p.m., 90 °C). After 3 h of deproteinization of the molts in sodium hydroxide, the obtained material was filtered and washed with deionized water on a laboratory sieve until the pH of the effluent was neutral (pH < 7.5) [14]. Then, the obtained chitin was dried in a laboratory dryer (105 °C). The final chitin (3.6 g DM) in the form of flakes with a diameter of 0.5–2.0 mm (CHM) was stored in a sealed polypropylene container.

### 3.2. Research on the Influence of pH on the Efficiency of Dye Sorption

The dry mass of 0.050 g CHM was weighed on a precision balance into each of the series of conical flasks (250 mL). Then, 100 mL of a 50 mg/L dye solution with pHs ranging from 2 to 11 was added to each of the flasks. The flasks were placed on a laboratory shaker (150 r.p.m., vibration amplitude 30 mm). After 120 min, samples of the solutions (10 mL) were collected into previously prepared polyethylene test tubes. The concentration of dyes in the test tubes was determined with the spectrophotometric method. The pH of the solutions after sorption was also tested. The test series for each dye was performed in triplicate.

### 3.3. Studies on the Kinetics of Dye Sorption

The dry mass of 1.00 g CHM was weighed on a precision balance into each of the series of beakers (2000 mL). Then, 2000 mL of a dye solution with concentrations of 50 and 250 mg/L (for RB5/ RY84/ BR64) or 10 and 50 mg/L (for BV10) and the optimal pH (determined as in Section 3.2). The beakers were placed on a multi-station magnetic stirrer (150 r.p.m.). After the specified times (0, 10, 20, 30, 45, 60, 90, 120, 150, 180, 210, 240, 270, 300, 330, and 360 min), samples of the solutions (5 mL) were taken from the beakers for spectrophotometric determination of the concentration of the dye remaining in the solution. The tests were performed in triplicate.

### 3.4. Research on the Maximum Sorption Capacity of CHM

The dry mass of 0.050 g CHM was added to each of the series of conical flasks (250 mL) on a precision balance. Then, dye solutions (100 mL) with concentrations ranging from 10 to 500 mg/L and the optimal pH for the tested dye (Section 3.2) were added to the flasks. The flasks were placed on a laboratory shaker (150 r.p.m., vibration amplitude 30 mm) for the sorption equilibrium time (determined as in Section 3.3). After the sorption had been completed, a sample (10 mL) was taken from each flask to determine the dye concentration. The tests were performed in triplicate.

### 3.5. Calculational Methods

The amount of dye that was adsorbed on the CHM was determined based on Equation (1):

Q_S_ = ((C_0_ − C_S_) × V)/m (1)

Q_S_ mass of sorbed dye [mg/g DM]
C_0_ initial concentration of dye [mg/L]
C_S_ concentration of dye after sorption [mg/L]
V volume of the solution [L]
m mass of the sorbent (CHM) [g DM].

The experimental data from the research on the kinetics of dye sorption on CHM were described using the pseudo-first-order model (2), pseudo-second-order model (3) as well as the intramolecular diffusion model (4).

q = q_e_ × (1 − e ^(−k1×t)^) (2)

q = (k_2_ × q_e_^2^ × t)/(1 *+* k_2_ × q_e_ × t) (3)

q = k_id_ × t^0.5^ (4)

q instantaneous value of sorbed dye [mg/g DM]
q_e_ the amount of dye sorbed at the equilibrium state [mg/g DM]
t time of sorption [min]
k_1_ pseudo-first order adsorption rate constant [1/min]
k_2_ pseudo-second order adsorption rate constant [g/(mg × min)]
k_id_ intramolecular diffusion model adsorption rate constant [mg/(g × min 0.5)]

Experimental data in studies on the maximum sorption capacity of CHM were described with three sorption isotherms: Langmuir 1 (5), Langmuir 2 (Langmuir double isotherm) (6), and Freundlich (7).

Q_S_ = (Q_max_ × K_C_ × C)/(1 + K_C_ × C) (5)

Q_S_ = (b_1_ × K_1_ × C)/(1 + K_1_ × C) + (b_2_ × K_2_ × C)/(1 + K_2_ × C) (6)

Q_S_ = K × C^n^ (7)

Q_S_ mass of sorbed dye [mg/g DM]
Q_max_ maximum sorption capacity in Langmuir equation [mg/g DM]
b_1_ maximum sorption capacity of sorbent (type I active sites) [mg/g DM]
b_2_ maximum sorption capacity of sorbent (type II active sites) [mg/g DM]
K_C_ constant in Langmuir equation [L/mg]
K_1_,K_2_ constants in Langmuir 2 equation [L/mg]
K the equilibrium sorption constant in Freundlich model
n Freundlich equilibrium constant
C concentration of the dye remaining in the solution [mg/L].

## 4. Results and Discussion

### 4.1. Characteristics of CHM (FTIR, Surface, SEM)

**Figure 1:**
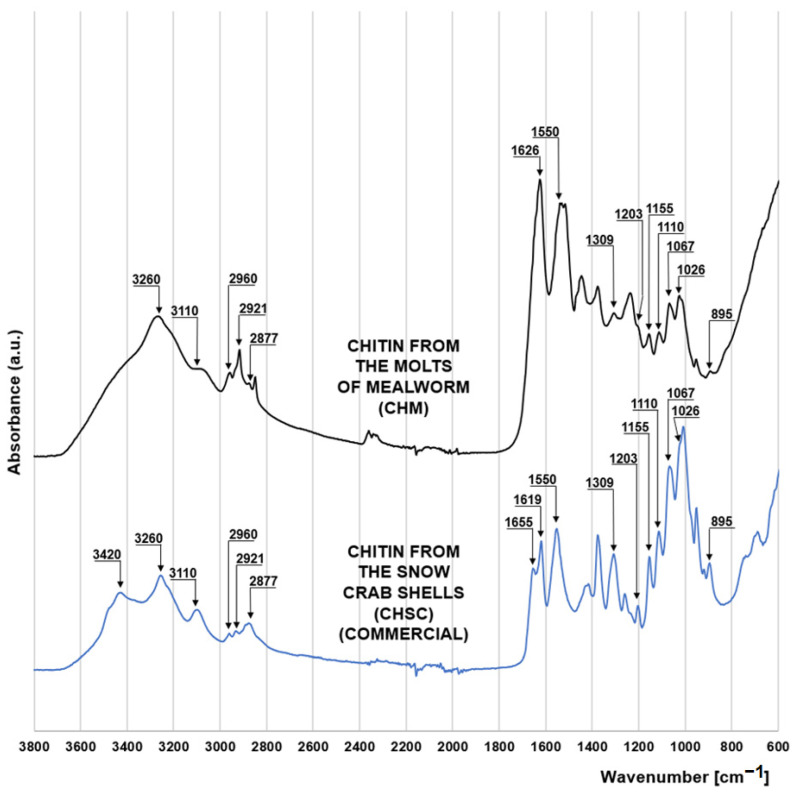
FTIR spectra for chitin from the molts of mealworm (CHM) and chitin from the snow crab shells (CHSC).

The FTIR spectrum of chitin from molts of mealworm (CHM) is very similar to that of the commercial chitin in the form of snow crab shell flakes (CHSC) (manufactured by BioLog Heppe GmbH, Landsberg, Germany) (Figure 1). Both materials show a series of peaks characteristic of saccharides. The peaks at 1067 cm^−1^ and 1026 cm^−1^ correspond to the stretches of the C-O bond of the C3 and C6 carbon of the pyranose (glucose) ring. Peaks at 1155 cm^−1^ and 1203 cm^−1^ indicate asymmetric and symmetric stretching of the C-O-C bond of the saccharide ring [15], while the peaks at 895 cm^−1^ and 1110 cm^−1^ indicate stretching of the saccharide ring due to the presence of the β-1-4 glycosidic bond in the structure of the polysaccharide [16,17]. The peaks at 2921 and 2877 cm^−1^, also characteristic of polysaccharides, can be attributed to the symmetric and asymmetric stretching of the C-H bond, respectively [18]. The presence of a methine group in the pyranose ring is evidenced by the peak at 2960 cm^−1^ [19]. The peaks characteristic of chitin at 1550 cm^−1^ and 1309 cm^−1^ indicate the bending of the N-H bond and stretching of the C-H bond of the acetamide functional group [20], respectively. Peaks at 3260 cm^−1^ and 3110 cm^−1^ are also associated with the acetamide functional group of chitin, indicating N-H bonding of amide I [19].

The spectrum of crab chitin (CHSC) has a distinct peak at 3420 cm^−1^, which is responsible for the N-H binding of the primary amine and is indicative of the presence of the amino group. The lack of this peak in the mealworm chitin spectrum may indicate a very low degree of its deacetylation. Characteristic for the CHSC spectrum are also the peaks at 1655 cm^−1^ and 1619 cm^−1^ corresponding to the stretching of the C=O bond of amide I. The peak at 1655 cm^−1^ indicates C=O stretching by the hydrogen bond with the N-H (acetamide group) of the adjacent chitin chain (CO-NH hydrogen bond), while the peak at 1619 cm^−1^ can be assigned to a specific C=O hydrogen bond with the hydroxymethyl group of the next chitin residue of the same chain (CO-HOCH_2_ hydrogen bond) [20]. The two discussed peaks suggest that CHSC has the crystalline form of α-chitin, which is typical of marine crustaceans. Contrary to the crab chitin spectrum, the mealworm chitin spectrum in the 1700–1600 cm^−1^ range has only one distinct peak at 1626 cm^−1^ indicating a hydrogen bond between the carbonyl groups of amide I and amide II of the same chain [21,22]. This may suggest that a significant part of the chitin coming from the mealworms has the form of β-chitin [23], typical of the highly elastic structures.

Using the Gemini VI apparatus from Micromeritics USA, the porous structure and pore distribution were measured for CHM and CHSC using the low-temperature nitrogen sorption method. The determined BET area for CHM was 4.524 m^2^/g and was similar to the area of snow crab chitin (2.486 m^2^/g). The pore diameter in the sorbent was quite small and averaged 1.797 nm. This indicates the microporous structure of the material. The existence of mainly micropores and a few mesopores in the CHM structure is also confirmed by the CHM pore distribution diagram (Figure S1a of the Supplementary Materials). Similar pore sizes were found for commercial CHSC (1.836 nm). The total pore volume determined for CHM was 0.00295 cm^3^/g (Figure S1b of the Supplementary Materials) and was more than twice as large as for CHSC (0.00115 cm^3^/g), which can be explained by the greater number of gaps.

**Figure 2:**
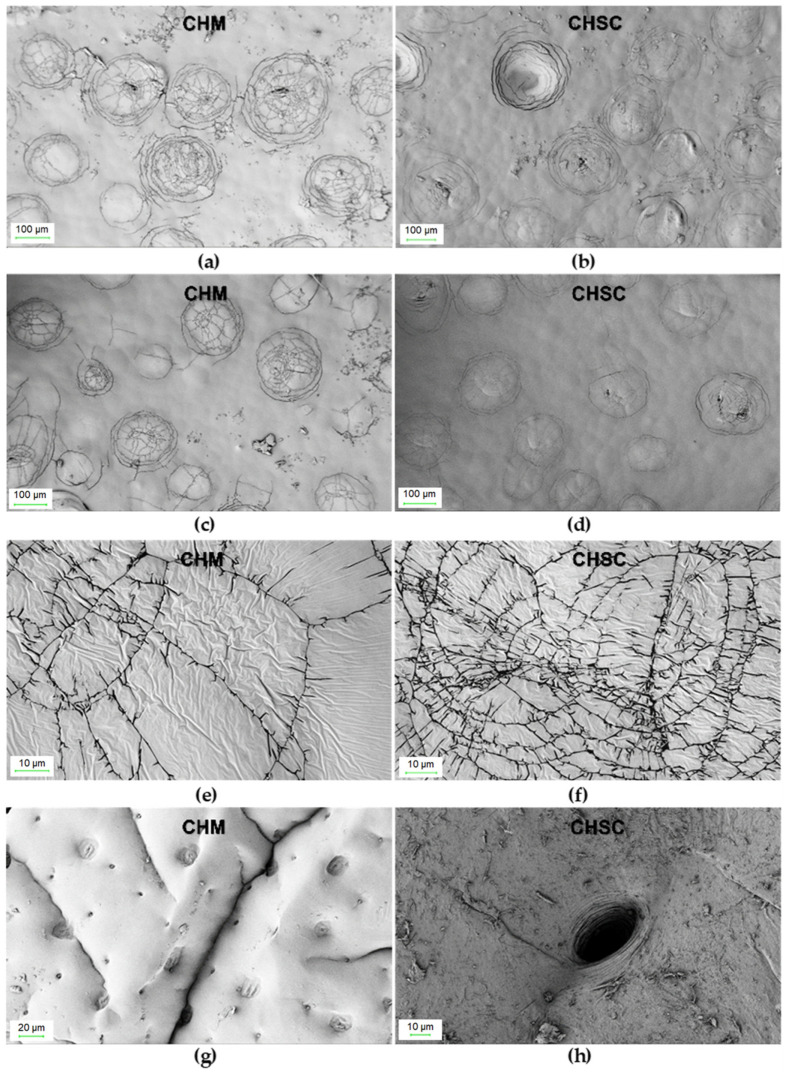
SEM images of: CHM (**a**,**c**,**e**,**g**) and CHSC (**b**,**d**,**f**,**h**) at different magnifications.

Surface morphology (SEM) images were taken for the CHM tested in the study and for the CHSN (Figure 2).

Chitin from mealworms has a morphology very similar to that of snow crab shells. In both cases, the surface is relatively little developed and the number of pores observed in the photos is low. As can be clearly seen in Figure 2c,d, there are clusters of circular fractures resembling a spider’s web on the relatively smooth surface. However, in the case of CHM, the number of surface “cracks” appears to be higher, which explains its greater specific surface area as well as the total pore volume.

### 4.2. Influence of pH on the Dye Sorption Efficiency on CHM

**Figure 3:**
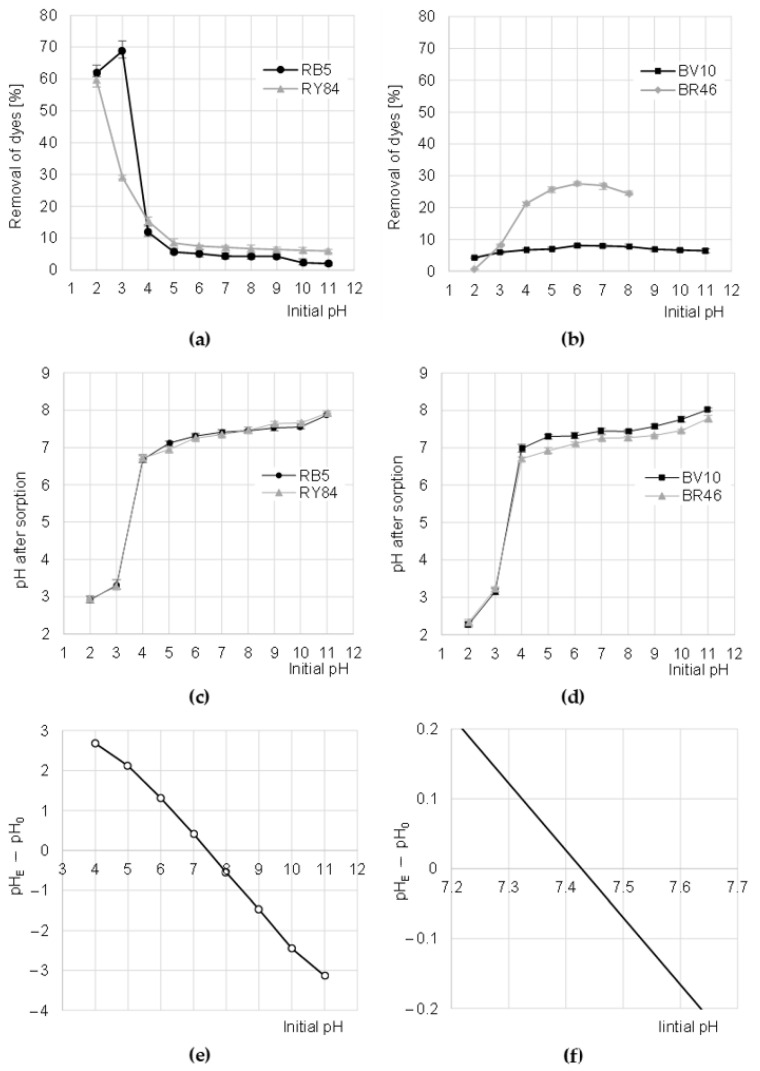
Effect of pH on the efficiency of sorption of: (**a**) anionic, and (**b**) cationic dyes onto CHM (average + range). Effect of CHM on changes in solution pH after sorption of: (**c**) Anionic and (**d**) cationic dyes. (**e**,**f**) Determination of pH_PZC_ of the CHM with the Boehm’s titration method. Temp. 22 °C.

The sorption efficiency of RY84 on CHM was the highest at pH 2, while for the RB5 dye—at pH 3 (Figure 3a). The increase in pH in the solution resulted in a decrease in the binding efficiency of anionic dyes to CHM, with the greatest decrease observed in sorption intensity at pH 3–5 (for RB5) and pH 2–5 (for RY84). The binding efficiency of RB5 and RY84 to CHM was the lowest at pH 11 (Figure 3a).

The high sorption efficiency of RB5 and RY84 at low pH was due to the basic acetamide functional groups of the sorbent as well as the anionic nature of the dyes. In the acidic environment (pH 2–3), with an excess of hydronium ions, there was an intense protonation of the acetamide functional groups CHM [24].

(−CH_2_-CO-NH_2_ + H_3_O^+^ → −CH_2_-CO-NH_3_^+^ + H_2_O)

The positively charged acetamide groups attracted electrostatically anions of the dyes, which in turn significantly enhanced their sorption. As the pH increased, the number of protonated acetamide groups in CHM decreased, which translated into successively decreasing efficiency of RB5 and RY84 binding. At pH > 4, the number of ionized acetamide groups of CHM was already low, which explains the generally low sorption efficiency of anionic dyes in the range of pH 5–11 (Figure 3a). At pH > 9, the sorption of dyes could be additionally limited by competition with OH− ions for sorption centers. The worse sorption efficiency observed in the case of RB5 at pH 2 than at pH 3 may result from the competition of the dye with a large amount of Cl^−^ ions. This effect was not observed in the case of RY84, presumably due to the high content of secondary amine groups in the dye structure (R1-NH-R2), which could bind excess Cl^−^ in the protonated form.

The positive effect of low pH on the sorption efficiency of anionic pigments was also noted in studies on RB5 sorption on snow crab chitin [25], egg shells [26], carbon nanotubes [27] as well as macadamia seed scales [28].

The sorption efficiency of cationic dyes BV10 and RB46 on CHM increased with pH increasing up to pH 6. A further increase of the initial pH value of dye solutions resulted in a step-wise reduction of dye binding efficiency on the tested sorbent (Figure 3b). The low efficiency of sorption of cationic dyes on CHM at low pH resulted from the acquisition of a strong positive charge by the sorbent, which electrostatically repelled the cations of basic dyes [29]. As mentioned earlier, increasing the pH of the solutions successively decreased the number of protonated functional groups of the sorbent. This translated into a gradually weaker positive charge on its surface, and consequently, into increasingly more effective sorption of BV10 and BR46. Presumably, at pH 6–7, the binding of cationic dyes on CHM took place mainly through hydrogen bonds (between hydrogen and nitrogen atoms as well as hydrogen and oxygen) as well as Van der Waals forces. In an alkaline environment, as a result of the excess of OH- ions in the solution, some of the hydroxyl groups of CHM could be deprotonated, owing to which the tested sorbent began to gain a negative charge (−OH + OH^−^→−O^−^ + H_2_O). However, despite the cationic nature of the dyes, the high pH had no positive effect on the sorption process, presumably because of the increasing concentration of Na^+^ cations in the solution, which competed with dye cations for free CHM sorption centers. The solutions of BR46 dye at pH > 8 are spontaneously discolored, which was confirmed in preliminary studies. Therefore, Figure 3b does not show the results of BR46 sorption at pH 9–11.

A similar effect of pH on the efficiency of BV10 sorption was noted in studies addressing the sorption of cationic dyes on aluminosilicates [30], fly ash and also soybean pomace [31]. In the case of BR46 sorption, a similar effect of pH was also obtained in the research on the decolorization of aqueous solutions on materials based on cyclodextrin [32], bentonite [33] and green tea leaves and coffee grounds [34].

During the sorption of dyes on CHM, the pH of the solutions changed, which is typical of the physical sorption processes. At the initial pH range of 4–11, the pH after sorption was adjusted to the pH range of 6.75–8.00 (Figure 3c,d). The mechanism of this process is as follows. At low pH, protons from hydronium ions bind (protonate) the functional groups of the sorbent, which in turn raises the pH of the solution. However, at high pH, some functional groups of the sorbent are deprotonated, and the resulting “free” hydrogen cations “neutralize” the hydroxide anions, while lowering the pH of the solution. The system always tends to the equilibrium state, i.e., the pH at which the number of positively charged functional groups of the tested sorbent is the same as the number of negatively charged groups. This pH is referred to as the zero charge point (pH_PZC_). The pH_PZC_ value determined for CHM with the Boehm titration method was 7.42 (Figure 3e). This confirms that the chitin flakes obtained from the molts of mealworms are slightly alkaline.

Further stages of the research were carried out at the pH values optimal for dye sorption (pH 3 for RB5, pH 2 for RY84 and pH 6 for BV10 and BR46).

### 4.3. Kinetics of the Sorption of Dyes on CHM

**Figure 4:**
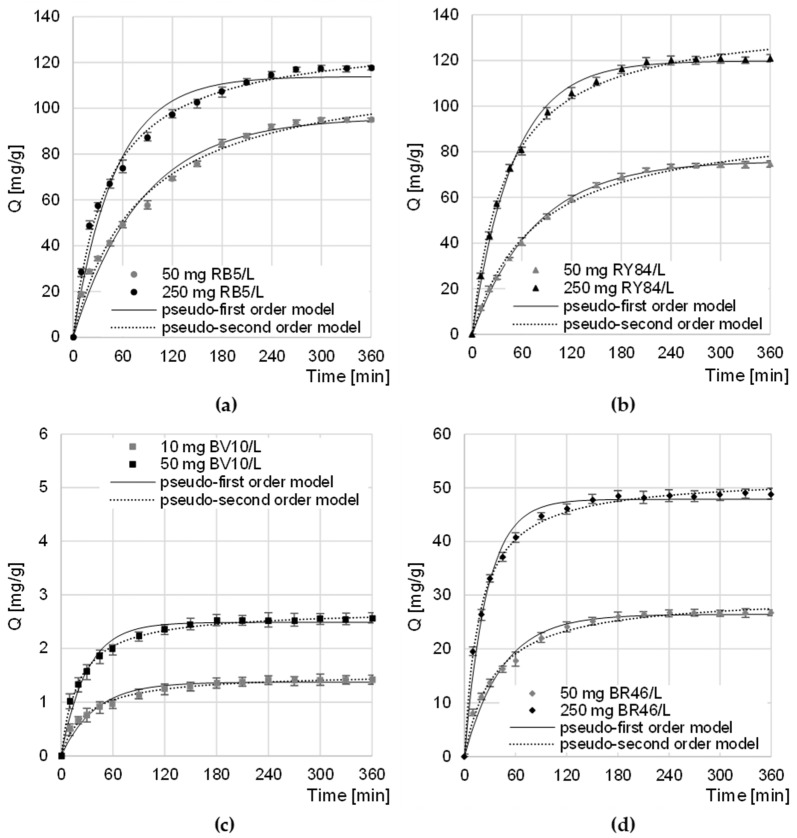
Changes in the concentration of: (**a**) RB5, (**b**) RY84, (**c**) BV10, and (**d**) BR46 during sorption onto CHM. Temp. 22 °C.

The equilibrium time of sorption of anionic dyes on CHM decreased slightly with increasing dye concentration to 270–300 min for RB5 and 240–270 min for RY84 (Figure 4a,b). The sorption intensity of RB5 and RY84 on CHM was the highest in the first minutes of the process. After 60 min, the amount of dye bound to the sorbent ranged from 52% to 63% of the q_e_ value (equilibrium amount of dye adsorbed on the sorbent) for RB5 and from 54% to 67% q_e_ for RY84. A similar sorption equilibrium time (360 min) was recorded in the research on the sorption of RB5 on activated carbon from walnut wood [35] and on chitin from snow crab shells [25].

The equilibrium time of BV10 and BR46 sorption on CHM, as in the case of anionic dyes, also depended on the initial concentration of the dye and ranged from 180 min (at the highest concentration) to 240 min (at the lowest initial concentration) (Figure 4c,d). Within the first 60 min of sorption, the amount of CHM-bound cationic dye ranged from 68.6 to 79.1% of the q_e_ value—in the BV10 test series and from 66.7 to 84.1% q_e_ in the BR46 test series. A similar time of BV10 sorption equilibrium was obtained in the research on the decolorization of water solutions on fly ash and soybean pomace (240 min) [31]. In the case of BR46, similar sorption equilibrium times were obtained during the research on the sorption of dyes on green tea leaves and coffee grounds (240 min) [34] as well as lemon peels (240 min) [36].

The shorter sorption times of dyes at their higher concentrations probably resulted from the greater probability of collisions of sorbent particles with sorbent active sites, which translated into faster saturation of sorption centers and completion of the process. Shorter sorption times recorded for cationic dyes than for anionic dyes could result, among others, from their much lower molar masses, which facilitated the penetration of the sorbent structure and faster occupation of the available sorption centers.

The obtained experimental data was described using the pseudo-first and pseudo-second-order models (Table 2, Figure 4). The values of the determination coefficients (R^2^) determined from the models indicate that, in each research series, regardless of the initial dye concentration, sorption was best described by the pseudo-secondary model. The obtained result is typical of the sorption of organic dyes on biosorbents [37,38,39].

The q_e_ values obtained in the test series with anionic dyes, despite significantly different initial dye concentrations, may indicate a relatively high affinity of sorbates (RB5 and RY84) to CHM active sites (Table 2). The sorption efficiency of cationic dyes, compared to anionic dyes, was much more dependent on their initial concentrations in the solution. This may suggest a low usefulness of CHM in the treatment of sewage containing low concentrations of basic dyes.

**Figure 5:**
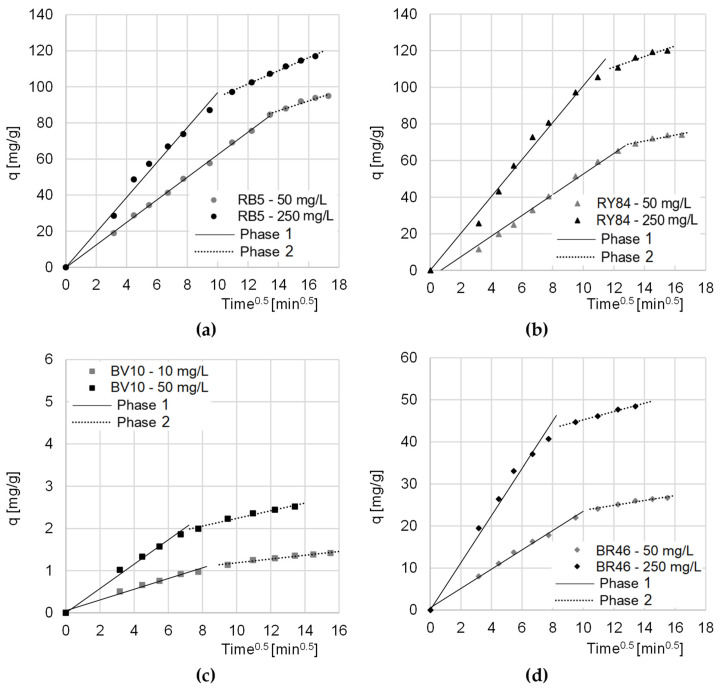
The intramolecular diffusion model of sorption of: (**a**) RB5, (**b**) RY84, (**c**) BV10, and (**d**) BR46 onto CHM. Temp. 22 °C.

Data from the research on the kinetics of dyes sorption on CHM was also described with the intramolecular diffusion model. The analysis of the constants determined from the given model showed that sorption on the tested sorbent, regardless of the type of dye and its initial concentration, occurred in two main phases (Table 3, Figure 5).

The first phase of sorption was the most intense. In this phase, the dye molecules diffused from the solution onto the surface of the sorbent, and the sorbate occupied the most accessible active sites. The second phase began when most of the active centers on the surface of the sorption material were saturated. In this phase, the dyes mainly occupied hard-to-reach sorption sites, located in the deeper layers of the sorbent. At this stage, there was an increased competition between dye molecules for the last free active sites. Due to the strongly limited number of remaining sorption sites, phase 2 featured a lower sorption intensity than phase 1 (k_d2_ < k_d1_) (Table 2). Phase 2 ended when the sorbent was no longer able to bind more dye molecules. The end of the second sorption phase also determined the moment of reaching the sorption equilibrium.

In the case of the sorption of anionic dyes, the relatively long period of the first phase indicated very good availability of CHM sorption centers. This had a positive effect on the sorption rate and indicated a high sorbent efficiency.

Compared to cationic dyes (BV10, RB46), anionic dyes (RB5, RY84) obtained significantly higher values of q_e_ (pseudo-second order model) (Table 2) and k_d1_/k_d1_ (intramolecular diffusion model) (Table 3). This indicates that CHM’s sorption capacity is much greater with respect to anionic than cationic dyes.

The noticeably higher sorption efficiency of BR46 on CHM compared to BV10 may be due to the lower molecular weight of Basic Red 46. The smaller sorbate particles had a greater ability to penetrate the chitin sorbent structure, and thus to occupy a greater number of sorption sites. The higher sorption efficiency of BR46 compared to BV10 could also result from having a greater number of amine groups and the possibility of creating more hydrogen bonds with CHM functional groups.

### 4.4. Maximum Sorption Capacity of CHM

**Figure 6:**
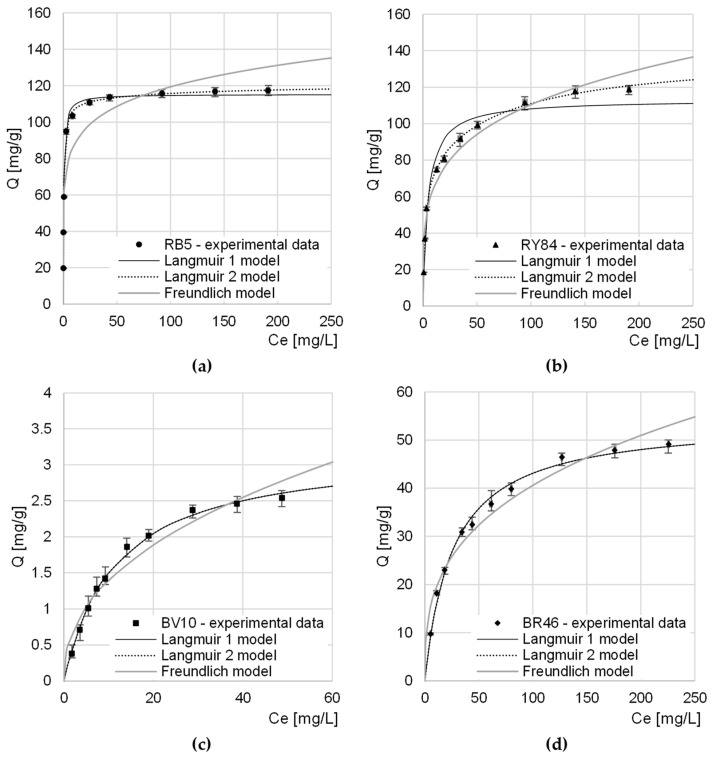
Isotherms of sorption of: (**a**) RB5, (**b**) RY84, (**c**) BV10, and (**d**) BR46 onto CHM. Temp. 22 °C.

Experimental data from research on the maximum sorption capacity of RB5 and RY84 by CHM was described with three popular sorption models: Langmuir 1 isotherm, Langmuir 2 isotherm, and Freundlich isotherm (Table 4, Figure 6).

In each research series, Langmuir models (1 and 2) showed a greater fit to the obtained data than the Freundlich model. This suggests that dye molecules formed a monolayer on the CHM surface and only one sorbate molecule could attach to one sorption center. However, it is possible for dyes to move within the monolayer and exchange between active sites.

Both in the case of the research series with RB5 and with RY84, the Langmuir 2 model described the experimental data better than the Langmuir 1 model did. This indicates a high probability of the involvement of at least two types of active centers in the sorption process. Presumably, in the case of CHM, the sorption sites in question were acetamide functional groups (described by the constants b_1_, K_1_) and amino groups (described by the constants b_2_ and K_2_). A few primary amine groups on the CHM surface could be formed as a result of the deacetylation of acetamide groups during the bath of mealworm molts in a sodium hydroxide solution.

In the case of cationic dyes, the Q_max_ and K_C_/K_1_/K_2_ constants determined from the Langmuir 1 and Langmuir 2 models as well as the coefficients of determination R^2^ had the same numerical values. This suggests that only 1 type of sorption center was involved in the sorption of BV10 and BR46 on CHM. Presumably, these centers are the nitrogen atoms in the acetamide groups of the chitin, which participate in the formation of hydrogen bonds with the amine groups of the cationic dyes.

The determined maximum sorption capacity of anionic dyes by CHM was similar and reached 121.15 mg/g and 138.55 mg/g for RB5 and RY84, respectively (Table 4, Figure 6). The relatively high anionic dyes sorption capacity of CHM is most likely due to a large number of basic functional groups capable of easy protonation (acetamide and amine groups), which are the key sorption centers for anionic impurities.

Due to the basic nature of CHM, its capacity for cationic dyes sorption was much lower than that for anionic dyes and reached 3.22 mg/g and 59.56 mg/g for BV10 and BR46, respectively. The greater sorption capacity of CHM in relation to BR46 as in comparison to BV10 may result from the much lower molar mass of the dye and also from the possibility of creating more hydrogen bonds with the sorbent centers of the tested sorbent.

The dye sorption capacities of CHM were compared with the sorption capacities of other unconventional sorbents and activated carbons (literature data—Table 5).

The sorption capacity of CHM in relation to BV10 is low compared to other sorbents based on plant biomass, such as: Fruit peels, leaves of crops, sawdust or seed husks (Table 5). The sorption properties of the sorbent tested in the work towards BV10 are similar to chitosan, which is a deacetylated derivative of chitin, and like chitin, has an alkaline character. In the case of BR46 sorption, CHM is fairly average compared to other sorbents. The sorbent tested in the study shows a better sorption capacity in relation to Basic Red 46 than sawdust-based sorbents, but it is also less efficient than such materials as nut shells, pine needles and cones, pumpkin seed husks or spent coffee grounds (Table 5). The list shows that the basic chitin-based materials are not the best sorbents for BV10 and BR46. It seems that materials of plant origin, usually acidic in nature, have much greater potential in the treatment of industrial wastewater from cationic dyes.

The sorption efficiency of anionic dyes RB5 and RY84 on CHM is many times higher than in the case of sorbents based on plant biomass, such as: Seed husks, stems of crops or sawdust. As mentioned above, the high sorption capacity of CHM towards anionic dyes is due to the basic nature of the biopolymer. The acidic nature of most of the sorption materials based on plant biomass significantly reduces the binding of anionic substances (Table 5).

CHM has a higher sorption capacity in relation to anionic dyes than some types of activated carbons (Table 5). This indicates a high usefulness of CHM in the treatment of wastewater containing anionic dyes.

The sorption capacity of mealworm molt chitin obtained in this study was very similar to the sorption capacity of high-quality chitin from snow crab shells, produced for the needs of the biomedical industry (Table 5). This may suggest that the mealworm molts are a comparably good source of chitin as the shells of sea crustaceans.

## 5. Conclusions

Chitin from mealworm molts is a very effective sorbent for RB5 and RY84 anionic dyes (Q_max_ = 121.15 mg/g and Q_max_ = 138.55 mg/g, respectively). CHM has comparable sorption properties to commercial chitin from snow crab shells as well as some types of activated carbons (literature data). The high capacity of CHM in relation to anionic dyes is due to its acetamide and amine functional groups responsible for its basic nature and also being the main sorption centers for most anionic compounds. However, the basic nature of CHM is the reason for its relatively poor sorption properties in relation to the cationic dyes BV10 and BR46 (Q_max_ = 3.22 mg/g and Q_max_ = 59.56 mg/g, respectively).

The sorption efficiency of dyes on CHM largely depends on solution pH. Sorption of anionic dyes is most effective at low pH (pH2 for RY84 and pH3 for RB5), while sorption of cationic dyes (BV10 and BR46) at pH 6.

CHM is potent to modify the pH of the solution during sorption. This is due to the fact that the system always tends to obtain a pH close to the pH_PZC_ value of the sorbent (CHM pH_PZC_ was 7.44).

The equilibrium time of sorption on CHM is 240 to 300 min for anionic dyes and 180 to 240 min for cationic dyes. Shorter sorption times are obtained at higher initial dye concentrations, which is presumably due to the greater likelihood of collisions of sorbent particles with sorbent active sites, resulting in faster saturation of sorption centers and termination of the process.

Sorption of dyes on CHM takes place in two main sorption phases, differing in intensity and duration. The dyes binding efficiency is the highest in the first key stage of the process.

At least two types of sorption centers play an important role in the sorption of anionic dyes (RB5 and RY84) on CHM. Presumably, the active sites in question are acetamide groups as well as amino groups (formed as a result of deacetylation of amino groups during the bath of mealworm molts in sodium hydroxide). Only one type of active site plays a key role in binding cationic dyes (BV10 and BR46) to CHM. Presumably, it is nitrogen atoms in the acetamide groups of chitin, which participate in the formation of hydrogen bonds with the amine groups of cationic dyes.

## Figures and Tables

**Table 1:** Characteristics of the dyes used in the study.

Dye	Reactive Black 5 (RB5)	Reactive Yellow 84 (RY84)	Basic Violet 10 (BV10)	Basic Red 46 (BR46)
Structural formula	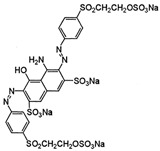	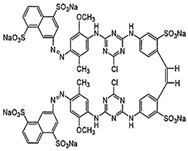	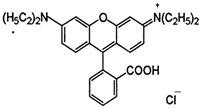	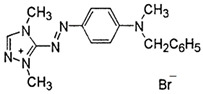
Molecular weight	991.8 g/mol	1628.2 g/mol	479.0 g/mol	321.4 g/mol
Dye class	double azo dye	double azo dye	xanthene dye	single azo dye
Dye type	anionic (reactive)	anionic (reactive)	cationic	cationic
λ_max_	600 nm	356 nm	554 nm	530 nm
Uses	Dyeing cotton, wool, and polyamide fibers	Dyeing polyester, cotton, rayon	Dyeing textiles, paper, leather	Dyeing leather, paper, wool, and acrylic fibers
Hazards	Irritating the respiratory tract, allergic reactions	Irritating the respiratory tract, possible allergic reactions	Toxic, fluorescent, carcinogenic; can induce skin and eye allergies	Caustic, toxic, hazardous to the aquatic environment
Other trade names	Begazol Black B, Diamira Black B, Remazol Black B	Active Yellow HE-4R, Apollocion Yellow H-E4R, Lamafix Yellow HER	Rhodamine B, Basic Red RB, Violet B	Cationic Red X-GRL, Anilan Red GRL, Sevron Fast Red GRL

**Table 2:** Kinetic parameters of sorption of dyes onto CHM determined from the pseudo-first order and pseudo-second order models.

Dye	Dye Conc.	Pseudo-First Order Model			Pseudo-Second Order Model			Exp. Data
		k_1_	q_e,cal._	R^2^	k_2_	q_e,cal._	R^2^	q_e,exp._
	[mg/L]	[1/min]	[mg/g]	-	[g/mg × min]	[mg/g]	-	[mg/g]
RB5	50	0.0120	95.83	0.9836	0.0001	119.01	0.9910	94.94
	250	0.0198	113.85	0.9766	0.0002	131.98	0.9953	117.56
RY84	50	0.0131	75.96	0.9942	0.0001	94.40	0.9943	74.69
	250	0.0201	119.76	0.9961	0.0002	139.30	0.9969	120.84
BV10	10	0.0256	1.38	0.9661	0.0224	1.55	0.9911	1.42
	50	0.0342	2.48	0.9770	0.0182	2.73	0.9964	2.56
BR46	50	0.0225	26.42	0.9849	0.0010	30.13	0.9932	26.71
	250	0.0385	47.86	0.9869	0.0011	52.13	0.9973	48.77

**Table 3:** Dye diffusion rate constants, determined from the intramolecular diffusion model. * [mg/(g × min^0.5^)].

Sorbent	Dye Conc.	Phase I			Phase II		
		k_d1_ *	Duration	R^2^	k_d2_	Duration	R^2^
	[mg/L]	[*]	[min]	-	[*]	[min]	-
RB5	50	6.254	180	0.9989	2.749	120	0.9625
	250	9.681	120	0.9967	3.654	150	0.9943
RY84	50	5.648	150	0.9912	1.629	120	0.8980
	250	10.082	120	0.9972	2.902	120	0.8659
BV10	10	0.127	60	0.9816	0.045	180	0.9654
	50	0.288	45	0.9979	0.091	135	0.9698
BR46	50	2.299	90	0.9958	0.577	150	0.9603
	250	5.607	60	0.9965	0.980	120	0.9885

**Table 4:** Constants determined from Langmuir 1, Langmuir 2, and Freundlich models.

Dye	Langmuir 1 Model			Langmuir 2 Model						Freundlich Model		
	Q_max_	K_c_	R^2^	Q_max_	b_1_	K_1_	b_2_	K_2_	R^2^	k	n	R^2^
	[mg/g]	[L/mg]	-	[mg/g]	[mg/g]	[L/mg]	[mg/g]	[L/mg]	-	-	-	-
RB5	115.29	1.784	0.9964	121.15	111.32	1.939	9.83	0.010	0.9985	63.74	0.136	0.8045
RY84	113.15	0.215	0.9494	138.54	75.63	0.538	62.91	0.014	0.9963	38.05	0.231	0.9482
BV10	3.22	0.087	0.9949	3.22	1.56	0.087	1.67	0.087	0.9949	0.51	0.43	0.9423
BR46	54.20	0.039	0.9910	54.20	27.81	0.039	26.39	0.039	0.9910	8.97	0.32	0.9550

**Table 5:** Comparison of the sorption capacity of different sorbents in relation to RB5, RY84, BV10, and BR46.

Dye	Sorbent	Q_max_ [mg/g]	pH	Time [min]	Source
RB5	Chitin flakes from snow crab shells (produced by BioLog Heppe)	131.56	3	360	[25]
	Activated carbon powder (high quality)	125.79	2	240	[40]
	Chitin from the molts of mealworm	121.15	3	300	This work
	Activated carbon modified with SPC	69.9	2	<60	[41]
	Chitin flakes from shrimp shells (produced by Sigma-Aldrich, St. Louis, MO, USA)	~60	6	~600	[42]
	Activated carbon (commercial. powder)	58.8	-	-	[43]
	Cotton stems	35.7	1	360	[44]
	Rape stalks	32.8	2.5	120	[45]
	Chitin flakes from snow crab shells (produced by BioLog Heppe, Landsberg, Germany)	30.37	4	180	[46]
	Wheat straw	15.7	7	195	[47]
	Beech sawdust	13.9	3	1440	[48]
	Cotton seed hulls	12.9	2	30	[49]
	Hen feathers	5.19	2	210	[50]
	Compost	4.80	3	120	[51]
	Macadamia seed husks	1.21	3	510	[28]
	Sunflower biomass	1.10	2	210	[52]
	Pumpkin seed husks	1.00	3	60	[53]
	Coconut shells	0.82	2	60	[54]
RY84	Chitin from the molts of mealworms	138.54	3	270	This work
	Algae biomass of *Chlorella* sp.	73.49	2	180	[55]
	Hydroxyapatite	50.25	5	180	[56]
	Activated carbon from the *Borassus flabellifer* plant	40.00	b.d	b.d.	[57]
	Sunflower seed husks	4.15	2	90	[58]
BV10	Commercial activated carbon powder	72.5	4	1440	[49]
	Activated carbon	30.0	3	-	[59]
	Beech sawdust	29.0	3	1440	[48]
	Coconut shells	28.5	3	180	[54]
	Jute fiber activated carbon	28.0	8	220	[60]
	Baker’s yeast	25.2	6.5	72	[61]
	Banana peel	20.6	6	1440	[62]
	Walnut shell fiber	18.7	–	–	[63]
	Coconut fiber	16.5	7	90	[64]
	Sugar cane fiber	10.4	–	–	[65]
	Chitosan (hydrogel beads)	5.79	4	1440	[48]
	Lemon peel	5.66	3	240	[36]
	Cedar cones	4.60	-	720	[66]
	Mango leaves (powder)	3.30	-	48	[67]
	Chitin from the molts of mealworms	3.22	6	120	This work
	Coffee powder	2.50	2	180	[68]
	Fly ash	1.90	-	72	[69]
BR46	Spent coffee grounds	179.4	6	240	[34]
	Pine cones	73.5	8	75	[70]
	Pine leaves	71.9	6	75	[71]
	Coconut shells	68.5	6	120	[54]
	Activated carbon from biomass of *Cerbera odollam*	65.7	7	90	[72]
	Chitin from the molts of mealworms	59.56	6	120	This work
	Lemon peel	54.0	6	240	[36]
	Rapeseed hulls	49.0	8	10	[73]
	Activated carbon ROW 08	45.0	8	60	[74]
	*Paulownia tomentosa* tree leaves	43.1	8	70	[75]
	Nut sawdust	30.1	7	-	[76]
	Sawdust	13.9	-	-	[77]

## Data Availability

Not applicable.

## Supplementary Materials

The following supporting information can be downloaded at: https://www.mdpi.com/article/10.3390/ma16020545/s1.
