# Supplementary material for: The Use of Chitin from the Molts of Mealworm (Tenebrio molitor) for the Removal of Anionic and Cationic Dyes from Aqueous Solutions
Source: Materials (Basel). 2023 Jan 5;16(2):545. doi: 10.3390/ma16020545 (PMC9865315; doi:10.3390/ma16020545)
Supplement: Supplementary file 1 [file materials-16-00545-s001.zip › materials-2082443-supplementary.pdf]

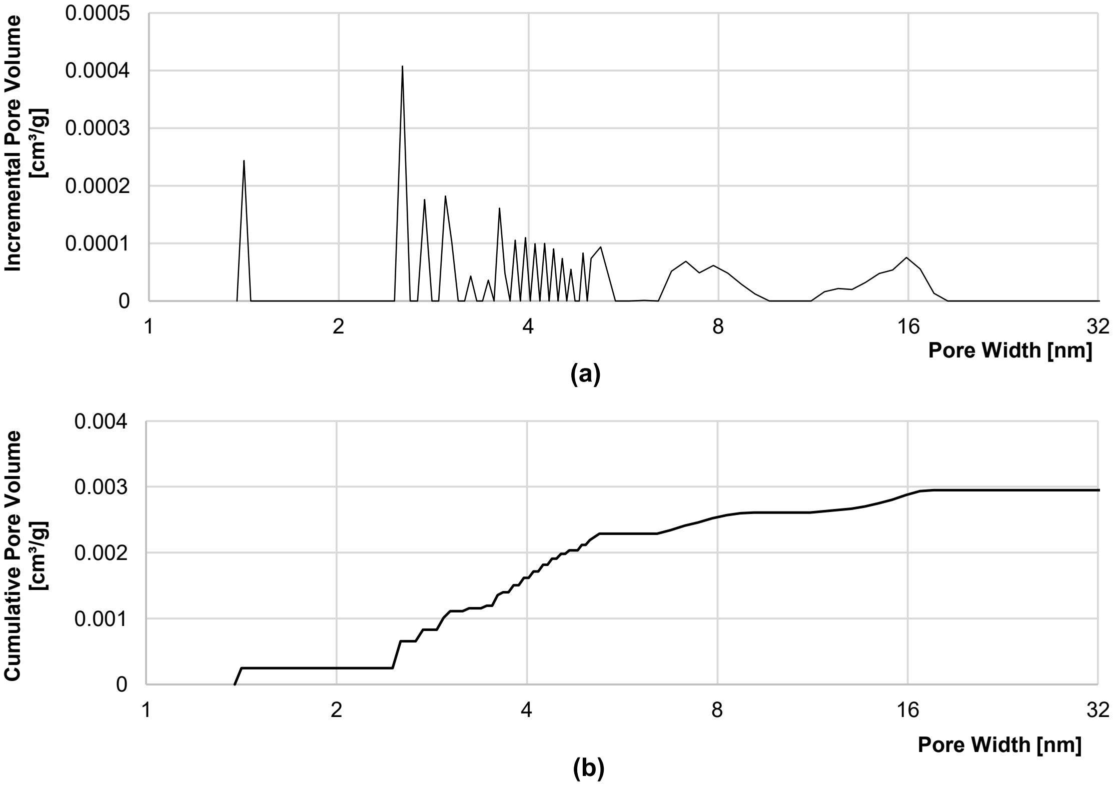

Figure S1. Pore distribution diagram for CHM. (a) Incremental pore volume versus pore diameter, (b) Incremental pore volume versus pore diameter.
